# Dental noise exposed mice display depressive-like phenotypes

**DOI:** 10.1186/s13041-016-0229-z

**Published:** 2016-05-10

**Authors:** Yujie Dong, Ying Zhou, Xixia Chu, Shiqing Chen, Lei Chen, Beimeng Yang, Xu Zhang, Lin Wang, Shuai Wang, Jingyu Lou, Qing Deng, Li Wang, Zheyi Cao, Jianan Wang, Jiaxin Xie, Tatiana Serdyuk, Shengtian Li, Lin He, Xiaoping Chen, Weidong Li

**Affiliations:** Bio-X Institutes, Key Laboratory for the Genetics of Development and Neuropsychiatric Disorders (Ministry of Education), Shanghai Key Laboratory of Psychotic Disorders, and Brain Science and Technology Research Center, Shanghai Jiao Tong University, 800 Dongchuan Road, Shanghai, 200240 China; Shanghai Elli Dental Clinic, No.26 South Yili Road, Shanghai, China; National Key Laboratory of Human Factors Engineering, China Astronaut Research and Training Center, Beijing, 100094 China

**Keywords:** Dental noise, Depression, Sucrose preference, Forced swimming, Fluoxetine, Neurogenesis, Weight

## Abstract

**Background:**

Studies have indicated that depressive disorders are observed frequently in dentists. It’s suggested that dentists encounter numerous sources of stress in their professional career. We noticed that the noises in dental environments are very unpleasant. The animal modeling studies suggested that stressful noise could produce depressive-like phenotypes in rodent animals. We hypothesize that the dental noise may be one of the primary stressors causing depressive disorders in dentists.

**Results:**

We treated C57BL/6 mice with programmatically played wide-spectrum dental noise for 8 h/day at 75 ± 10 dB SPL level for 30 days, and then tested the behaviors. After exposure to dental noise, animals displayed the depressive-like phenotypes, accompanied by inhibition of neurogenesis in hippocampus. These deficits were ameliorated by orally administered with antidepressant fluoxetine.

**Conclusions:**

Our results suggested that dental noise could be one of the primary stressors for the pathogenesis of depressive disorders and the dental noise mouse model could be used in further depression studies.

**Electronic supplementary material:**

The online version of this article (doi:10.1186/s13041-016-0229-z) contains supplementary material, which is available to authorized users.

## Background

Studies have indicated that depressive disorders are observed frequently in dentists than other professional groups [1, 2]. It has been considered that dentists encounter numerous sources of stress including feeling physically or emotionally exhausted, headaches or backaches, coping with difficult or uncooperative patients, heavy workload and financial problems [3] in their professional career. Consequently, mental disorders such as anxiety, depression and even suicide may result from these stresses. The suicide rate among dentists was much higher than that of other occupations according to the death data of 21 states of USA in late 20th century, and the suicide rate of dentists is 4.45–5.43 times more than general working-age population according to different logistic regression analysis methods [4]. All these evidences revealed that dentists suffer from the stressful work.

Dentists are predisposed to a number of occupational hazards such as viral infection, dental materials, radiation, noise and eyestrain [5]. We noticed that noises in dental environments are very unpleasant. Noise pollution has been criticized by mankind for decades of years and was historically regarded as a large public concern [6]. The inner ear hair cell system of rodents was very similar to that of human beings and other mammals [7]. The animal modeling studies suggest that stressful noise might produce neurogenesis impairment in rodent animals [8–10]. Increasing evidences implied that impaired hippocampal neurogenesis is intimately linked with the onset of depression as discussed in the neurogenic hypothesis of depression [11], so the decrease of dentate gyrus neurogenesis is considered a causal factor for depression disorder [12].

Wrap these all together, we hypothesize that the dental noise may be one of the primary stressors causing depressive disorders in dentists. So we attempted to establish a dental noise-exposed mice model to test and verify whether dental noise could produce depressive-like phenotypes in mice.

## Methods

### Animals

Adult male C57BL/6 mice (10 weeks of age) were from Charles River Laboratories. The mice were housed in standard cages with the cycle of 12 h light to 12 h dark (light from 7:00 am to 7:00 pm), with 40–50 % relative humidity and temperature 24 ± 2 °C. All animal experiments were approved by Institutional Animal Care and Use Committee (IACUC) of Shanghai Jiao Tong University.

### The dental noise

**Fig. 1:**
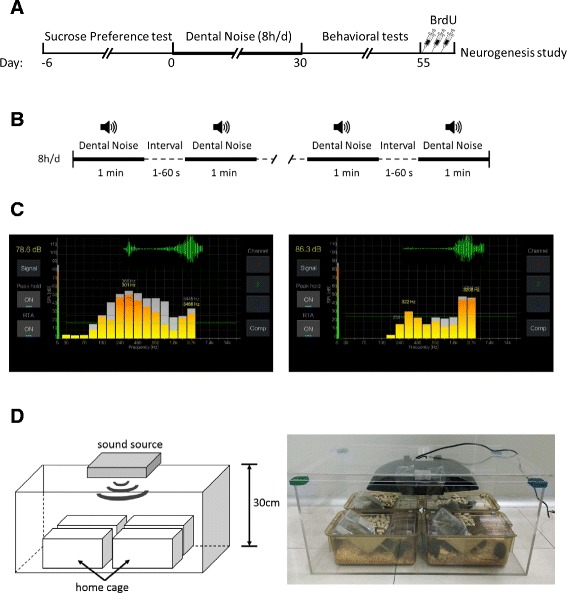
Experimental design. **a** The timeline of this experiment. **b** The stress regimen during the establishment period: one of the recorded two types of noises was played randomly for 1 min in a loop with random intervals from 1 to 60 s, lasting 8 h every day for totally 30 days. **c** Sound pressure level (SPL) and sound frequency spectrum analysis in real time of two noises by SPL Spectrum Analyzer Software. The *bar charts* represent wide spectrum distribution and the *green waveform* represents the wave crests at around 300 Hz and 3 kHz. *Left*: noise type 1, the high speed turbine dental drill; *Right*: noise type 2, the ultrasonic tooth cleaner. **d** *Left*: The experimental apparatus used to play the noise to animals. Mice were placed in home cages inside a plexiglass box, 30 cm from sound source on the top. *Right*: Photos of the real experimental setup

Two types of noises in this experiment were recorded from Shanghai Elli Dental Clinic, noise type 1 is from the high speed turbine dental drill and noise type 2 is from the ultrasonic tooth cleaner. The noises had wide-spectrum frequency (wave crests at 300 Hz and 3 kHz) and were played by the audio device JBL MS202 WT as sound source (Fig. 1c). Animals were placed in home cages inside a plexiglass box, 30 cm from sound source (Fig. 1d).

These two types of dental noise from Shanghai Elli Dental Clinic in this study are available in the Additional file 1.

### Dental noise mouse model and treatment

Mice were exposed to noise from a dental clinic (Fig. 1a). Dental noise (75 ± 10 dB SPL) lower than the reversible threshold of tissue injury [13] was played 8 h/d (from 9:00 am to 5:00 pm) for 30 days with a program mode of 1 min looped noise with random intervals from 1 to 60 s (Fig. 1b). The mice were administered orally with 120 mg/L fluoxetine hydrochloride (Flu, Jinhchem, Shanghai, China) in water (Noise/Flu) or water only (Noise/Veh) during 30 days. Fluoxetine should be available and renewed every day to ensure efficacy. Mice with (Con/Flu) or without (Con/Veh) fluoxetine hydrochloride were used as control groups absent from the exposure to dental noise.

### Weight change

All the mice were weighed every 10 days during the experiment. Define D0 the starting day of giving noise, and the weighing time was at D-10, D0, D10, D20, D30 and D40. The body weight increase rate was recorded and compared among four groups. The increase rate from D(m) to D(n) was calculated as follows.

$$ \mathrm{Increase}\kern0.5em \mathrm{rate}\frac{\mathrm{weight}\kern0.5em \mathrm{D}\left(\mathrm{n}\right)-\mathrm{weight}\kern0.5em \mathrm{D}\left(\mathrm{m}\right)}{\mathrm{weight}\kern0.5em \mathrm{D}\left(\mathrm{m}\right)}\times 100\% $$

### Sucrose preference test

Sucrose preference task is commonly used for evaluating the depressive-like phenotypes in animals. Sucrose preference is defined as the ratio of the consumption of sucrose solution and the consumption of both pure water and sucrose solution. All the mice underwent the Sucrose Preference test before and after the noise exposure stage.

The procedures were conducted as follows: The mice were singly housed in a quiet room without any other disruptors. Before the test, mice were trained to adapt to drinking sucrose solution. Two bottles were prepared for each mouse containing pure water and 2 % sucrose solution, respectively. The positions of two bottles were exchanged every 24 h. After 3 days of habituation, carry on the water/sucrose consumption test after 17 h of food and water deprivation. Mice were given a bottle of pure water and a bottle of 2 % sucrose solution weighed in advance. Those two bottles were weighed 1, 12 and 24 h after resuming water. The consumption of sucrose solution, pure water and total water was recorded. Sucrose preference was calculated as follows, and mice with sucrose preference under 65 % were considered as anhedonia.

$$ \mathrm{Sucrose}\kern0.5em \mathrm{preferene}\frac{\mathrm{sucrose}\kern0.5em \mathrm{solution}\kern0.5em \mathrm{consumption}}{\mathrm{total}\kern0.5em \mathrm{water}\kern0.5em \mathrm{consumption}\left(\mathrm{pure}\kern0.5em \mathrm{water}+\mathrm{sucrose}\right)}\times 100\% $$

### Open field test

The Open field test was utilized to examine locomotor activity and anxious behavior. Every mouse was placed in a square plexiglass box (27.5 cm L× 27.5 cm W × 18 cm H) and was allowed to explore the arena freely for 20 min. The total distance of movement was recorded by software of Med Associates inc.

### Elevated plus-maze test

The apparatus consisted of four arms (29 cm L × 8 cm W) at 90° angles to each other. At each trial, the mouse was placed in the center with its nose directed toward the closed arm and was allowed to explore the maze freely for 5 min. The entry frequency and stay time in the open arms and closed arms was calculated, respectively.

### Sociability test

The apparatus consisted of a 3-chambered plexiglass box (60 cm L × 40 cm W × 50 cm H) divided by plexiglass walls with openings allowing animals to move between chambers. Small cages (8 cm diameter × 10 cm H) were placed in the two outer chambers for snout contact but not fighting between animals. Every mouse was first released in the central chamber and was allowed to freely explore the three chambers for a 10 min habituation period. A male wild-type stimulus mouse about 4–5 weeks old was then placed in one of the small cages and the experimental mouse was allowed to explore the apparatus for an additional 5 min. Time spent in each chamber and in sniffing of each cages was recorded.

### Forced swimming test

Forced Swimming test is an effective detection method for depression, and it is widely used for screening anti-depression drugs and testing drug efficacy [14]. Each mouse was individually placed in a plexiglass cylinder (20 cm H × 14 cm diameter) filled with 15 cm depth of water at 24 ± 1 °C for 5 min. Take mice out of water and clean them up after test. Record the latency to the first bout of immobility and calculate the total duration of immobility of the last 3 min. It could be judged immobility when all active movements stopped for over 2 s such as struggling and swimming, only floating or making minimal movements [15].

### Prepulse inhibition test

The test aimed to examine startle response as well as the function of sensorimotor gating. The session had a total of 90 trials by The SR-LAB™ Startle Response System. To evaluate the startle response, each of the first 10 trials is consisted of a 40 ms 120 dB “pulse alone” startle stimuli. The rest of the 80 trials are consisted of random delivery of: 20 “pulse alone” startle stimuli, 30 “pre” stimuli (at 76, 79 and 85 dB), and 30 “pre-pulse” trials that consisted of a single 120 dB pulse preceded by a 20 ms pre pulse. PPI was calculated as follows.

$$ \mathrm{P}\mathrm{P}\mathrm{I}\frac{\mathrm{Average}\kern0.5em V \max \kern0.5em ``\mathrm{p}\mathrm{ulse}\kern0.5em \mathrm{alone}"-\mathrm{Average}\kern0.5em V \max \kern0.5em ``\mathrm{p}\mathrm{r}\mathrm{e}\hbox{-} \mathrm{pulse}"}{\mathrm{Average}\kern0.5em V \max \kern0.5em ``\mathrm{p}\mathrm{ulse}\kern0.5em \mathrm{alone}"}\times 100\% $$

### Contextual fear conditioning test

Contextual Fear Conditioning test was performed in test boxes from Med Associates inc. This test consisted of a training phase and a testing phase. During the training phase, mice were individually placed in test boxes and were allowed to explore for 5 min with three 0.75 mA electric foot shock delivered from the floor of each box. Mice were then returned to their home cages. Twenty-four hours after training, mice were placed back to test boxes for another 5 min and the freezing time was recorded. Freezing was defined as the absence of all movement except for respiration.

### BrdU assay for neurogenesis

Mice were injected with 5′-Bromo-2-deoxyuridine (BrdU) (10 mg/ml; Sigma) of 100 mg/kg body weight in distilled water every 2 h for three times. Two hours after the last injection, mice were sacrificed and fresh brains were perfused and fixed in 4 % paraformaldehyde (Sigma) at 4 °C overnight. Transfer the brains into 30 % sucrose (Sigma) in phosphate buffered saline (PBS) until sunken. Cryosections at 40 μm for immunofluorescence were obtained with a cryostat CM 3080S (Leica).

After being incubated with blocking solution containing 5 % goat serum (Millipore) and 0.3 % Triton X-100 (Sigma) for 1 h at room temperature, sections were incubated in the primary antibody, rat anti-BrdU (Abcam) diluted 1:500, overnight at 4 °C in a humidified box. The sections were then rinsed in PBS and incubated with the secondary antibody, goat anti-rat IgG (Life Technologies) diluted 1:500, for 2 h at room temperature. At last, nuclei were labeled with fluorescent dye 4′-6-diamidino-2-phenylindole (DAPI) (10 μg/ml; Sigma). The number of BrdU-positive neurons in the granule cell layer (GCL) and the subgranular zone (SGZ) of dentate gyrus was counted under a a Leica confocal microscope.

### Statistical processing methods

We used unpaired two-tailed *t*-test and ANOVAs to compare different groups. Interactions between conditions (noise and control) and treatment (Flu and vehicle) were analyzed by two-way ANOVA followed by appropriate post-hoc tests using StatView Software. *P* < 0.05 indicates statistical significance between groups (*p < 0.05, **p < 0.01, ***p < 0.001), and all results are presented as mean ± SEM. Graphs were drawn by GraphPad Prism Software.

## Results

### Dental noise decreased body weight gain, which was ameliorated by antidepressant fluoxetine

**Fig. 2:**
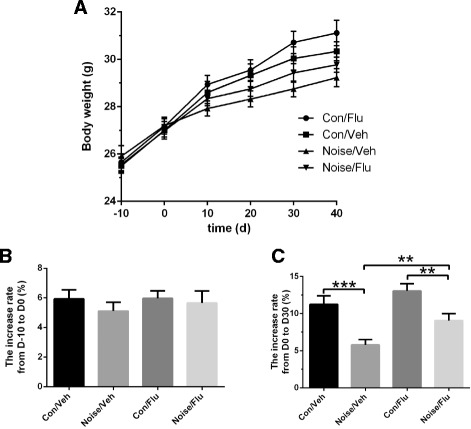
Effects of dental noise stress and fluoxetine on body weight. **a** The body weight from D-10 to D40 of the control group (Con/Veh), the control group administrated with fluoxetine (Con/Flu), the noise-exposed group (Noise/Veh) and the noise-exposed group with fluoxetine (Noise/Flu). **b** The increase rate of body weight among four groups from D-10 to D0. **c** The increase rate of body weight among four groups from D0 to D30, *p < 0.05,**p < 0.01, ***p < 0.001

A reduction in body weight was repeatedly associated with chronic stress [16]. To examine whether dental noise affects weight, we recorded the body weight throughout the experiment. It was exhibited in the body weight line chart that the Noise/Veh group had the lowest increase speed in weight than the three other groups during the exposure period from D0 to D30 (Fig. 2a; repeated ANOVA, F(9,171) = 6.206, *p* < 0.0001). There was no statistical significance on body weight of four groups during 10-day period before starting the noise exposure (Fig. 2b). However, after 30 days of dental noise stress, the Noise/Veh group had significantly low increase rate in body weight compared with the Con/Veh group, and fluoxetine treatment (Noise/Flu) could significantly elevate the weight increase rate comparing to the Noise/Veh group (Fig. 2c; Con/Flu *n* = 16, Con/Veh *n* = 15, Noise/Flu *n* = 15, Noise/Veh *n* = 15; two-way ANOVA, effect of condition: F(1,57) = 24.299, *p* < 0.0001; effect of treatment: F(1,57) = 7.073, *p* < 0.05; interaction: F(1,57) = 0.584, *p* = 0.448. Unpaired two-tailed *t*-test, Noise/Veh vs Con/Veh, *t* = 4.014, *p* < 0.001; Noise/Flu vs Noise/Veh, *t* = −2.792, *p* < 0.01). These results indicated that dental noise would lower the increase rate of body weight in mice and antidepressant could ameliorate it.

### Dental noise induced depressive-like phenotypes, which were reversed by fluoxetine

**Fig. 3:**
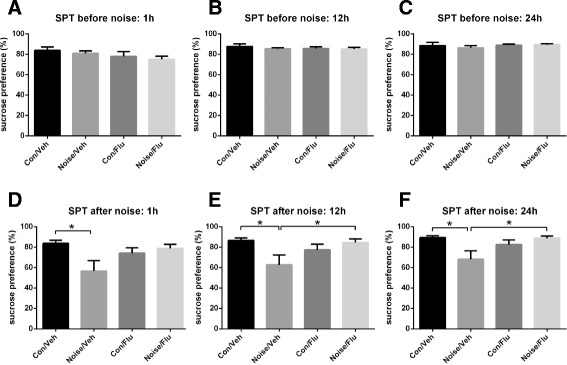
Effects of noise stress and fluoxetine on Sucrose Preference test. **a** Sucrose consumption before 30 days of noise. The sucrose preference in 1 h after food and water deprivation. **b** Sucrose consumption before 30 days of noise. The sucrose preference in 12 h after food and water deprivation. **c** Sucrose consumption before 30 days of noise. The sucrose preference in 24 h after food and water deprivation. **d** Sucrose consumption after 30 days of noise. The sucrose preference in 1 h after food and water deprivation. *p < 0.05. **e** Sucrose consumption after 30 days of noise. The sucrose preference in 12 h after food and water deprivation. *p < 0.05. **f** Sucrose consumption after 30 days of noise. The sucrose preference in 24 h after food and water deprivation. *p < 0.05

Next, we examined the impact of dental noise on the behaviors related to depression. Anhedonia, disrupted reward processing, is a core symptom of depressive disorders [17]. Sucrose Preference test was commonly used for evaluating the degree of anhedonia. Before being exposed to noise, there was no significant difference in sucrose preference among four groups in 1, 12 and 24 h (Fig. 3a, b and c) after 17 h of food and water deprivation. Following 30 days of dental noise exposure, sucrose preference decreased significantly for the Noise/Veh group in 1, 12 and 24 h after resuming water. With continuous fluoxetine administration, the Noise/Flu group performed a trend of increase in sucrose preference compared with that of the Noise/Veh group (Fig. 3d, e and f; Con/Flu *n* = 14, Con/Veh *n* = 13, Noise/Flu *n* = 13, Noise/Veh *n* = 13; two-way ANOVA, interaction; F(1,49) = 6.509, *p* < 0.05; F(1,49) = 6.963, *p* < 0.05; F(1,49) = 7.81, *p* < 0.01. Unpaired two-tailed *t*-test, Noise/Veh vs Con/Veh, *t* = 2.558, *p* < 0.05; *t* = 2.492, *p* < 0.05; *t* = 2.555, *p* < 0.05; Noise/Flu vs Noise/Veh, *t* = 2.033, *p* = 0.0532; *t* = 2.157, *p* < 0.05; *t* = 2.444, *p* < 0.05; separately in 1, 12 and 24 h). These results indicated that dental noise would induce depressive-like sucrose preference deficits which could be reversed by fluoxetine.123.

**Fig. 4:**
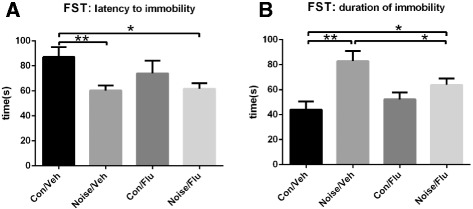
Effects of noise stress and fluoxetine on Forced Swimming test. **a** The latency to immobility among four groups. The Noise/Veh group presented significantly shorter latency to immobility than the Con/Veh group, while was no difference between the Noise/Flu group and the Noise/Veh group. *p < 0.05, ** *p* < 0.01. **b** The duration of immobility of the last 3 min among four groups. It was longer in the Noise/Veh group than the Con/Veh group and was decreased in the Noise/Flu group comparing with the Noise/Veh group after fluoxetine administration. *p < 0.05, ** *p* < 0.01

The Forced Swimming test is widely used for evaluating depressive-like phenotypes. The Noise/Veh group presented obvious variation to the Con/Veh group with shorter latency to immobility and longer duration of immobility. With fluoxetine administration, the Noise/Flu group performed significantly decreased duration of immobility compared with the Noise/Veh group, while there was no difference in the latency to immobility of these two groups (Fig. 4a; Con/Flu *n* = 15, Con/Veh *n* = 13, Noise/Flu *n* = 15, Noise/Veh *n* = 14; two-way ANOVA, effect of condition: F(1,53) = 7.501, *p* < 0.01; effect of treatment: F(1,53) = 0.661, *p* = 0.4197; interaction: F(1,53) = 1.031, *p* = 0.3145. Unpaired two-tailed *t*-test, Noise/Veh vs Con/Veh, *t* = 3.108, *p* < 0.01. Fig. 4b; two-way ANOVA, effect of condition: F(1,53) = 16.53, *p* < 0.001; effect of treatment: F(1,53) = 0.892, *p* = 0.3491; interaction: F(1,53) = 5.02, *p* < 0.05. Unpaired two-tailed *t*-test, Noise/Veh vs Con/Veh, *t* = −3.851, *p* < 0.01; Noise/Veh vs Noise/Flu, *t* = 2.16, *p* < 0.05). It suggested that dental noise induced depressive-like forced swimming impairment and fluoxetine treatment could improve it.

**Fig. 5:**
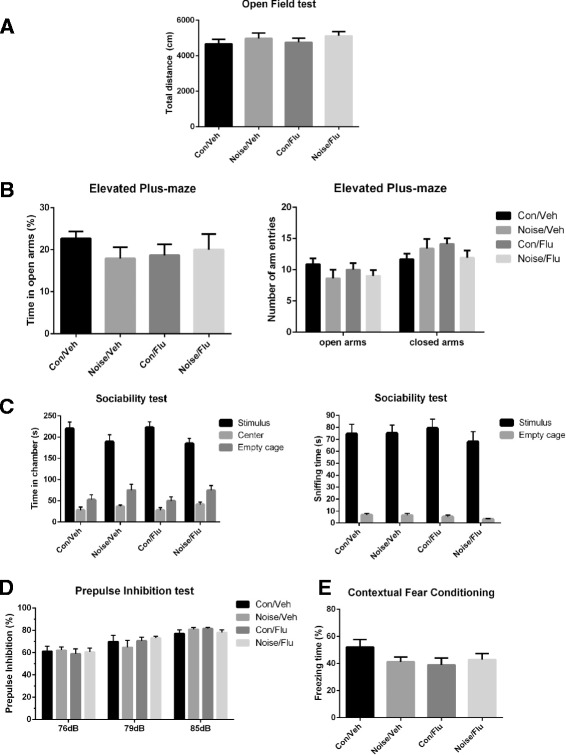
Results of other behavioral tests. **a** The total distance of movement in the Open Field test for 20 min. **b** Results of the Elevated Plus-Maze test. *Left*, percentage of time spent in the open or closed arms; *right*, percentage of entries into the open or closed arms. **c** Sociability test in the three-chambered apparatus. *Left*, the time spent in three chambers; *right*, the time spent in sniffing two cages. **d** Prepulpse Inhibition test of acoustic startle at prepulse intensity of 76, 79 and 85 dB. **e** Percentage of freezing time in the Contextual Fear Conditioning test

We also examined the mice in other behavioral tests. No significant difference was observed and no effect of fluoxetine treatment was revealed in the Open Field test (Fig. 5a), the Elevated Plus-Maze test (Fig. 5b), the Sociability test (Fig. 5c), the Prepulse Inhibition test (Fig. 5d) and the Contextual Fear Conditioning test (Fig. 5e) among four groups.

### Dental noise decreased hippocampal neurogenesis, which was reversed by fluoxetine

**Fig. 6:**
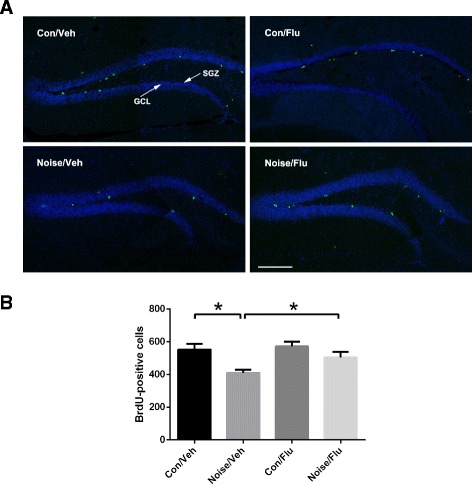
Effects of noise stress and fluoxetine on neurogenesis. **a** Confocal images of BrdU-positive neurons. Sections were immunofluorescent double-labeled for BrdU (*green*) and DAPI (*blue*). *Scale bar* indicates 200 μm. **b** The number of BrdU-positive neurons in the hippocampus after 30 days of noise treatment. The number was counted in the granule cell layer (GCL) and the subgranular zone (SGZ). *p < 0.05, **p < 0.01

It has been reported that depression is related with the neurogenesis decrease [18]. To identify whether dental noise affects neurogenesis, we examined the neural proliferation of hippocampus. BrdU was injected every 2 h for three times. Two hours after the last injection, mice were sacrificed for immunofluorescence. The number of BrdU-positive neurons in GCL and SGZ of dentate gyrus was counted under a Leica confocal microscope (Fig. 6a). Compared with the Con/Veh group, the Noise/Veh group exhibited significant decrease of BrdU-positive neurons after 30 days of noise stress. Fluoxetine treatment increased the number of BrdU-positive neurons of the Noise/Flu group in contrast to Noise/Veh group (Fig. 6b; Con/Flu *n* = 4, Con/Veh *n* = 4, Noise/Flu *n* = 5, Noise/Veh *n* = 4; two-way ANOVA, effect of condition: F(1,13) = 12.161, *p* < 0.01; treatment: F(1,13) = 4.3, *p* = 0.0585; interaction: F(1,13) = 1.955, *p* = 0.1855. Unpaired two-tailed *t*-test, Noise/Veh vs Con/Veh, *t* = 3.594, *p* < 0.05; Noise/Flu vs Noise/Veh, *t* = −2.779, *p* < 0.05). The result indicated that dental noise decreased hippocampal proliferation and fluoxetine could reverse it.

## Discussion

Higher suicide rate and risk of depressive disorders are observed frequently in dentists. Explanations for the phenomenon were widely discussed, which generally focused on occupational stress or sociodemographic factors, such as gender and divorce [19]. However, there was no study about the impact of dental noise per se. Strong noise was commonly believed to disturb normal life, causing mood irritability and even inducing exacerbate psychiatric disorders such as depression [20]. To assay the effect of dental noise on the depression, we established a dental noise exposed depressive-like mouse model. Dental noise exposure decreased the body weight increase rate, impaired the sucrose preference and prolonged the duration of immobility in Forced Swimming test, which were common features of depression. We did not observe the difference in other behavioral tests, such as Elevated Plus-Maze, Open Field test, Sociability Test, Prepulse inhibition test and Fear Conditioning test. It suggested that dental noise had specificity of inducing depressive-like phenotypes.

Fluoxetine, a selective serotonin (5-HT) reuptake inhibitor, was the most commonly prescribed treatment for depression [21]. Fluoxetine increases the concentration of extracellular 5-HT and makes the desensitization of presynaptic 5-HT1A receptors [22]. The depressive-like phenotypes induced by dental noise were evidently ameliorated with fluoxetine in the Sucrose Preference test and the Forced Swimming test. Fluoxetine administration also led to an increase in body weight in our experiment. Fluoxetine was reported to have anorectic effect as a serotonin reuptake inhibitor [23–25], this fluoxetine-induced weight gain argued that the antidepressant effect of fluoxetine overroded the anorectic effect [26]. In this experiment, fluoxetine was administrated from the beginning of dental noise exposure and it reduced the incidence of depressive-like phenotypes, so it was suggested that fluoxetine had protective effects from the impairment induced by dental noise.

Recently, increased studies suggested that adult hippocampal neurogenesis was associated with mental disorders including depression [27, 28], so we counted new-born neurons in dentate gyrus. The number of BrdU-positive neurons was decreased in Noise/Veh group comparing to Con/Veh, which hinted that dental noise negatively affects adult hippocampal proliferation. After fluoxetine administration, proliferation was significantly increased in the Noise/Flu group comparing to Noise/Veh. However, there was no change in proliferation of Con/Flu group comparing with Con/Veh group. Several studies have shown that chronic fluoxetine treatment increased the survival of BrdU-positive cells in wild type animals [29–31]. And studies also showed no significantly increased cells proliferation in mice after chronic fluoxetine treatment [31–33]. The effects of chronic fluoxetine treatment on survival and proliferation during adult hippocampal neurogenesis may need further studies.

There is still the possibility that our findings might not be restricted to the dental noise. Unpleasant noises from crowded traffic or other workplaces may have analogous influence, which could be studied in the future. Furthermore, sleep disturbances were associated with mental disorders [34]. The 8-h noise exposure might also affect the sleep of mice which might contribute to depressive-like phenotypes. It also needs to be investigated in our future studies.

Nevertheless, this is the first reported depressive mouse model induced by dental noise per se. However, further studies need to be performed to reveal the molecular mechanism of how dental noise affects neurogenesis and depression in our model.

## Conclusions

In conclusion, our results provide evidence that mice exposed to dental noise exhibit depressive-like phenotypes in behavior tests and neurogenesis. This is the first report demonstrating that dental noise could be one of the primary stressors for the pathogenesis of depressive disorders. The dental noise mouse model could be used in further depression studies.

### Ethics approval and consent to participate

All animal experiments were approved by Institutional Animal Care and Use Committee (IACUC) of Shanghai Jiao Tong University.

### Consent for publication

Not applicable.

### Availability of data and materials

The dental noise used in the experiment is available in Microsoft OneDrive. https://onedrive.live.com/redir?resid=3E89C4A9A8D4332F!460&authkey=!AHySn7wfIE5_uxg&ithint=folder%2cwav. Accessed 23 Mar 2016.

## Additional file

Additional file 1: Supplementary material. (PPTX 18,329 kb)

## Abbreviations

SPL: sound pressure level
Flu: fluoxetine
Veh: vehicle
PPI: Prepulse Inhibition
BrdU: 5′-Bromo-2-deoxyuridine
PBS: phosphate buffered saline
DAPI: 4′-6-diamidino-2-phenylindole
GCL: granule cell layer
SGZ: subgranular zone
