# Supplementary figures and images for: Dental noise exposed mice display depressive-like phenotypes
Source: Mol Brain. 2016 May 10;9:50. doi: 10.1186/s13041-016-0229-z (PMC4894364; doi:10.1186/s13041-016-0229-z)

## Slide 1
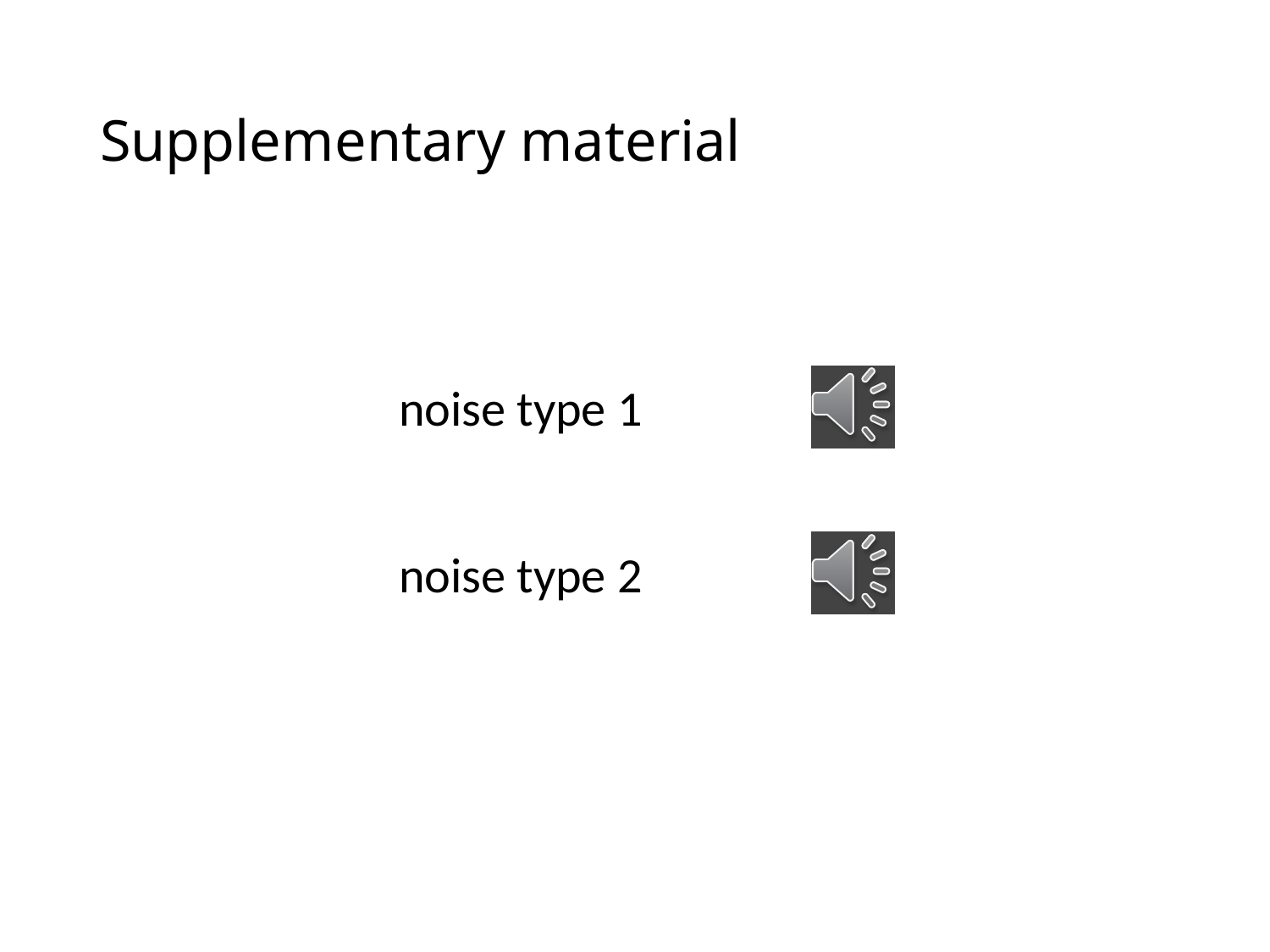

# Supplementary material
noise type 1
noise type 2

Supplement: Supplementary file 1 — Supplementary material. (PPTX 18,329 kb) [file 13041_2016_229_MOESM1_ESM.pptx]
